# Removal of an abluminal lining improves decellularization of human umbilical arteries

**DOI:** 10.1038/s41598-020-67417-4

**Published:** 2020-06-29

**Authors:** Ho-Yi Tuan-Mu, Yi-Hao Chang, Jin-Jia Hu

**Affiliations:** 1 Department of Physical Therapy, Tzu Chi University, Hualien, Taiwan; 2 Department of Sports Medicine Center, Hualien Tzu Chi Hospital, Buddhist Tzu Chi Medical Foundation, Hualien, Taiwan; 3 Department of Biomedical Engineering, National Cheng Kung University, Tainan, Taiwan; 4 Department of Mechanical Engineering, National Chiao Tung University, #1001 University Rd., Hsinchu, 300 Taiwan

**Keywords:** Biomedical engineering, Tissue engineering, Tissues

## Abstract

The decellularization of long segments of tubular tissues such as blood vessels may be improved by perfusing decellularization solution into their lumen. Particularly, transmural flow that may be introduced by the perfusion, if any, is beneficial to removing immunogenic cellular components in the vessel wall. When human umbilical arteries (HUAs) were perfused at a transmural pressure, however, very little transmural flow was observed. We hypothesized that a watertight lining at the abluminal surface of HUAs hampered the transmural flow and tested the hypothesis by subjecting the abluminal surface to enzyme digestion. Specifically, a highly viscous collagenase solution was applied onto the surface, thereby restricting the digestion to the surface. The localized digestion resulted in a water-permeable vessel without damaging the vessel wall. The presence of the abluminal lining and its successful removal were also supported by evidence from SEM, TEM, and mechanical testing. The collagenase-treated HUAs were decellularized with 1% sodium dodecyl sulfate (SDS) solution under either rotary agitation, simple perfusion, or pressurized perfusion. Regardless of decellularization conditions, the decellularization of HUAs was significantly enhanced after the abluminal lining removal. Particularly, complete removal of DNA was accomplished in 24 h by pressurized perfusion of the SDS solution. We conclude that the removal of the abluminal lining can improve the perfusion-assisted decellularization.

## Introduction

Atherosclerosis is the primary cause of vascular disease worldwide, resulting in heart attacks, stroke, and peripheral arterial disease^1,2^. Heart disease, most commonly due to atherosclerotic coronary disease, is the first cause of death in the US^3^. Balloon angioplasty with or without stenting has been regularly performed to open up affected arteries. In the case of peripheral arterial occlusion or severe narrowing, however, by-pass surgery may be preferred or required to restore blood flow and preserve functions of downstream tissues. Although synthetic vascular grafts made of ePTFE or Dacron have been successfully used for the reconstruction of large-diameter blood vessels, small-diameter synthetic grafts usually suffer from early-stage complications. Autologous arteries or veins remain the gold standard for small-diameter bypass grafts, which, however, are not always available. Tissue engineering potentially offers a solution for the lack of grafts in small-diameter bypass surgery.

Decellularized tissue scaffolds, among a variety of scaffolding materials used in tissue engineering, have shown promise in creating tissue substitutes including tendons^4^, bones^5^, nerves^6^, heart valves^7^, and blood vessels^8,9^. Decellularization is a process to remove immunogenic cellular components from native tissues or organs, while minimizing the associated adverse effects on the chemical composition, biological activity, and mechanical properties of their extracellular matrix (ECM). Except for thin tissues, the process is, in general, time consuming, and usually requires a combination of physical, chemical and enzymatic treatments to achieve satisfactory results^10^. Decellularized tissue scaffolds are essentially composed of ECM components and exhibit excellent biocompatibility^11–13^. As the ECM therein is largely preserved after decellularization, less efforts are required for recreating the composition and microstructure of native tissues, which are the results of a series of biological processes during the development and maturation of the tissue^14^. Recently, it has been found that non-chemically cross-linked decellularized tissues, when implanted, are able to modulate host response toward a constructive tissue remodeling^15^.

Particularly because of their advantageous mechanical properties, decellularized native blood vessels have been used as a scaffold to create tissue-engineered vascular grafts (TEVGs)^11,16,17^. Many xenogeneic^9,18^ and allogeneic^19,20^ blood vessels have been decellularized for this purpose. Specifically, decellularized human umbilical arteries (HUAs), which have an inner diameter less than 6 mm, represent an attractive scaffold for preparing small-diameter TEVGs because they are widely available and sufficiently long grafts without branches can be easily prepared.

For a general decellularization procedure, tissues to be processed are immersed in a decellularization solution and subjected to some form of agitation. The agitation increases the concentration of the decellularization agent and reduces the concentration of released cellular components at the tissue surface, facilitating the diffusion of the decellularization agent and cellular components into and out of the tissue, respectively. The agitation, however, has little influence on the interstitial transport within the tissue. That is, the effectiveness of agitation-based decellularization is determined mainly by passive diffusion of molecules^21^. Our previous study on decellularizing HUAs used short segments of HUAs that can be well decellularized by rotary agitation^22^. The rotary agitation may not be effective in delivering the decellularization solution into the lumen of the HUAs that are long enough for bypass surgery. Also, the diffusion of cellular components out of a long vessel may be limited to the abluminal side of the vessel.

In an effort to more consistently improve decellularization of human umbilical veins (HUVs), McFetridge’s group, instead of relying on agitation, perfused the vein at an elevated transmural pressure^21^. The improved decellularization efficiency was attributed to the transmural convective flow induced by the pressurized perfusion^21^. Supposedly, transmural flow in the vessel wall, if any, is beneficial to removing immunogenic cellular components therein. When we attempted to decellularize long segments of HUAs using a similar setup, however, very little transmural flow was observed. We hypothesized that a watertight lining at the abluminal side of HUAs hampered the transmural flow and tested the hypothesis by subjecting the abluminal surface of HUAs to enzyme digestion. Specifically, a highly viscous collagenase solution was applied onto the abluminal surface, thereby restricting the digestion to the surface. The vessel permeability, mechanical properties, and microstructure of collagenase-treated HUAs were examined in comparison with those of untreated HUAs in order to demonstrate the presence of the abluminal lining. Furthermore, both collagenase-treated and untreated HUAs were decellularized with 1% sodium dodecyl sulfate (SDS) solution under either rotary agitation, simple perfusion, or pressurized perfusion. Decellularization efficiency was examined by histology and residual DNA quantification over time.

## Materials and methods

### Preparation of human umbilical arteries

Human umbilical cords were obtained from the AN-AN Women and Children Clinic (Tainan, Taiwan) with patients’ consents and all the experimental protocols and the inform consent form were approved by the institutional review board/ethics committee of National Cheng Kung University Hospital (ER-99-060). Immediately after delivery, the cords were placed in cold Hank’s buffer saline solution and transferred to the laboratory for processing. Intact HUAs were isolated from the cords on ice using blunt dissection. Each HUA was cut in half; one half directly underwent decellularization or mechanical testing and the other half was subjected to the abluminal lining removal prior to further processing or testing. All methods were carried out in accordance with relevant guidelines and regulations.

### Enzymatic digestion of the abluminal surface

**Figure 1:**
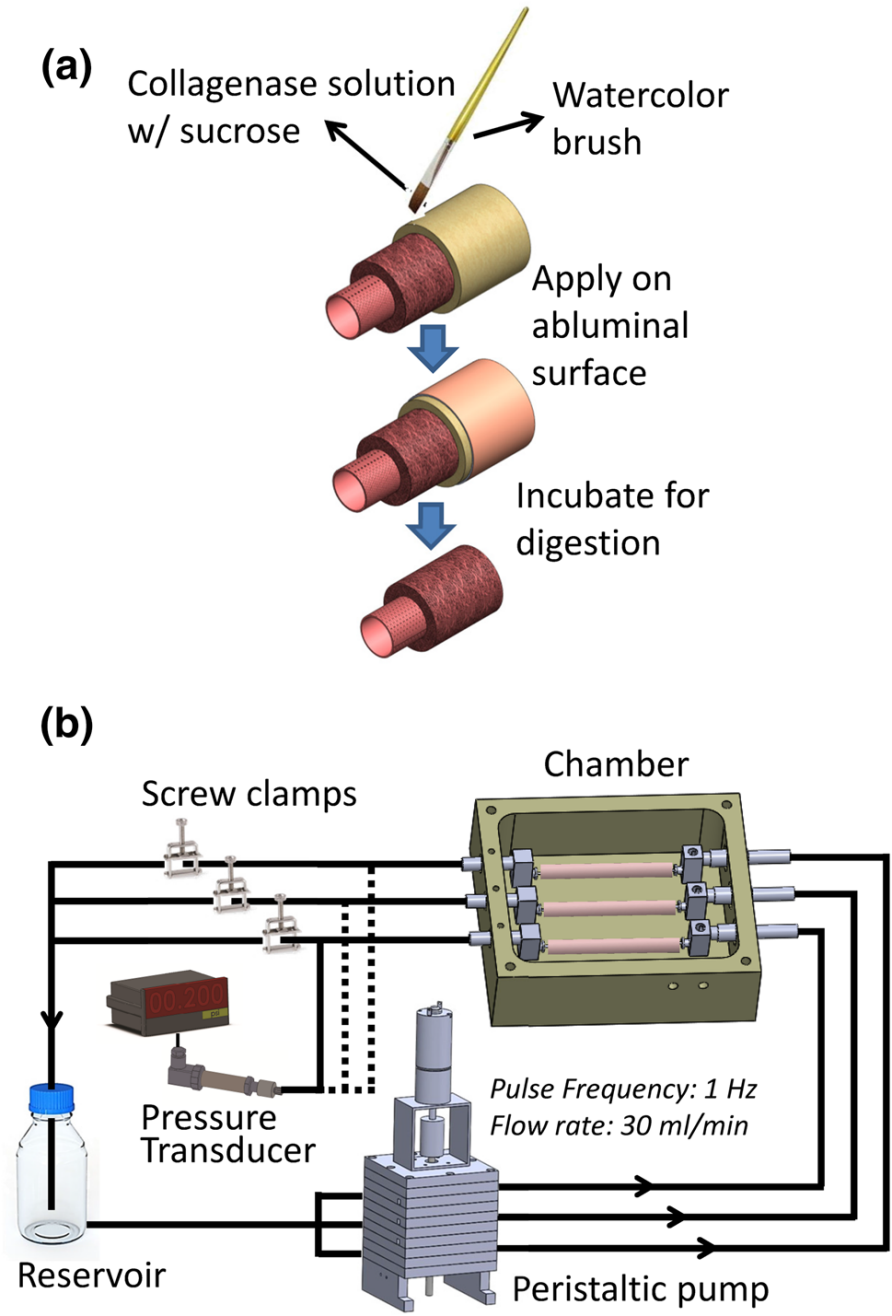
Schematics of the collagenase treatment procedures for removing the abluminal lining of HUAs (**a**) and of the perfusion system for decellularizing HUAs (**b**).

The abluminal surface of HUAs was treated with collagenase solutions of different viscosity (Table 1) to remove the abluminal lining. Prior to the treatment, a silicone tube was inserted into the lumen of the HUA to maintain its tubular shape. Both ends of the HUA were then cannulated with plugged Luer adaptors to prevent the collagenase solution from entering the luminal space. For those HUAs that were to be treated with the collagenase solution of low viscosity, the HUA was immersed and incubated therein at 37 °C in a humidified CO_2_ incubator for 30, 60 or 120 min. For those HUAs that were to be treated with the collagenase solution of medium or high viscosity, the collagenase solution was applied evenly onto the abluminal surface of the HUA to ensure that enzyme digestion occurred from the surface (Fig. 1). The HUA was then incubated at 37 °C in a humidified CO_2_ incubator for 30, 60 or 120 min. After the treatment, the collagenase was removed by washing with phosphate buffer. In this study, the HUA treated with the collagenase solution of high viscosity for 60 min was designated as Col-HUA. The HUA that was not treated with collagenase solutions (a control group) was designated as Unt-HUA.

**Table 1:** Compositions of collagenase solutions of different viscosity.

Collagenase conc. (%)	Collagenase^a^ (mg)	Sucrose (g)	HBSS (ml, final volume)	Viscosity (centipoise, cP)
0.1	10	0	10	Low viscosity ~ 1.0
0.2	20			
0.1	10	3	10	Medium viscosity ~ 2.8
0.2	20			
0.1	10	5	10	High viscosity ~ 7.5
0.2	20			

^a^Collagenase type I (CLS-1, Worthington, Lakewood, NJ) was used.

### Permeability of the vessel wall

For permeability testing, HUAs were first cannulated with Luer adaptors at both ends. Upon expelling air in the lumen, one end of the HUA was plugged and the other end was connected to a bottle filled with deionized water. The HUA was then pressurized to 30 cmH_2_O by raising the water level in the bottle. Transmural flow was measured with transmural pressure fixed at 30 cmH_2_O. The permeability ($k$) of the HUA vessel wall was calculated using Darcy’s law, which describes the fluid flow through porous media as $k = \frac{QL\mu }{{A_{s} \Delta P}}$, where $A_{s}$ is the luminal surface area of the HUA (m^2^), $\Delta P$ is the pressure drop across the vessel wall (mmHg), $\mu$ is the viscosity of deionized water (Pa s), $Q$ is the transmural flow (m^3^/s), and $L$ is the wall thickness measured by a custom-made high frequency ultrasound (lateral/axial resolution = 180/42 μm).

### Scanning electron microscopy

Scanning electron microscopy (SEM) was employed to examine the surface topography of Col-HUAs and Unt-HUAs cross-section. The fractured cross-section of the HUA was prepared by breaking the vessel in liquid nitrogen. The HUA was rinsed with PBS buffer, fixed in 2.5% glutaraldehyde solution for 1 h, rinsed again with deionized water, and then dehydrated with graded alcohols. Dried specimens were sputter-coated with platinum (Sputter E-1045, Hitachi, Japan) and viewed using a scanning electron microscope (S-4100, Hitachi, Japan).

### Transmission electron microscopy

Transmission electron microscopy (TEM) was employed to reveal the ultrastructure of Col-HUAs and Uni-HUAs cross-section near the abluminal surface. The HUA was rinsed with PBS buffer, fixed in 2.5% glutaraldehyde solution overnight at 4 °C, rinsed again with deionized water, and then dehydrated with graded alcohols. The dried specimens were prepared into ultra-thin sections and stained with uranyl acetate. Images were acquired by a transmission electron microscope (JEM-1400, JEOL Ltd., Japan).

### Decellularization

SDS was selected as the decellularization agent to evaluate the effect of the abluminal lining removal on the decellularization of HUAs^22^. SDS (Mallinckrodt Baker, Phillipsburg, NJ) was dissolved in deionized water to prepare 1% (w/v) SDS solution. Both Col-HUAs and Unt-HUAs were decellularized with 1% SDS solution under three decellularization conditions (i.e., rotary agitation, simple perfusion, and pressurized perfusion) for 12, 24, or 48 h. For the simple agitation group, the HUA (~ 6 cm) was immersed in the SDS solution on an orbital shaker at 100 rpm at room temperature^22^. For the simple perfusion and pressurized perfusion groups, the HUA (~ 6 cm) was cannulated with luer adapters using 5-O suture and coupled to a custom-built perfusion system, which consists of a peristatic pump, a chamber accommodating the vessels to be decellularized, a solution reservoir, a pressure transducer (Model 80A-005G, 0–5 psi, Sensormate, Taiwan) and screw clamps (Fig. 1). During decellularization, 1 L of the SDS solution was continuously circulated at an average flow rate of 30 mL/min and a pulse frequency of 1 Hz at room temperature. The transmural pressure was set at 30 ± 10 mmHg (± 10 was caused by pulse flow) by adjusting the screw clamp in the flow loop for the pressurized perfusion group whereas the screw clamp was not engaged for the simple perfusion group. The pressure was selected based on our pilot study in which significant condensation of the ECM was observed in the Col-HUAs perfused at a transmural pressure greater than 30 mmHg. After the specified period of time, specimens of 2 mm length were cut at least 1 cm away from the sutured ends, washed in deionized water with agitation at least five times until no bubbles were found in the water to remove residual SDS, and then processed for either histology or DNA quantification.

### Histology and image quantification

The specimens were fixed in 10% neutral-buffered formalin overnight at room temperature. After standard processing, the HUA was embedded in paraffin to enable examination of cross sections. Five micron sections were cut using a microtome (Leitz 1512, Leica, Germany) and collected on positively charged slides. The sections were stained with H&E, Alcian blue, and picro-sirius red (PSR) for illustration of nuclei, glycosaminoglycans (GAGs), and collagen, respectively. Histological images were acquired by an optical microscope (DM2500P, Leica, Germany) equipped with a CCD camera (DFC295 digital camera, Leica, Germany). Specifically, the PSR-stained sections were imaged under polarized light. Ten 24-bit color images of 2048 × 1563 pixel resolution were acquired under polarized light and normal light, respectively, for each specimen with a 40 × objective and saved in TIFF for semi-quantification of collagen and GAG. Relative collagen content was analyzed by a LabVIEW routine previously developed for quantifying collagen in PSR-stained sections^23^. Relative GAG content was analyzed similarly by another LabVIEW routine.

### DNA quantification

Quant-iT PicoGreen dsDNA assay kit (Invitrogen, USA) was used to quantify residual DNA in the specimen. Similar to the previously published protocol^22^, the specimen was lyophilized at − 40 °C for 24 h and its dry weight was measured. The dried specimen was then incubated in a papain solution, which contained 20 U/mL papain (Worthinton, Lakewood, NJ), 1.1 mM EDTA (Panreac, Spain), 5.5 mM cysteine-HCl (Panreac, Spain) and 0.067 mM 2-mercaptoethanol (Alfa Aesar, England) overnight at 60 °C until the HUA was completely digested. The solution was diluted with 0.2 M Tris–EDTA buffer and then incubated with the working solution of the kit in a 96-well plate. The fluorescence of the sample was measured using a fluorometer (excitation: 485 nm, emission: 538 nm; Fluoroskan Ascent, Thermo Fisher Scientific, Waltham, MA) and compared with that of a λ dsDNA standard (0–10 ng/mL) to determine the weight of residual DNA in the HUA. Finally, the weight of the residual DNA was normalized by the dry weight of the specimen.

### Mechanical testing of HUAs

The mechanical properties of Unt-HUAs, Col-HUAs, and decellularized Col-HUAs were evaluated by pressure-diameter tests using a custom-built mechanical tester^24^. Five HUAs were used; one from each of five donors. Every HUA was cut into three ~ 25-mm segments for preparing the three kinds of HUAs. First, the vessel was cannulated with Luer adaptors and then coupled to the loading frame of the tester. Specifically, every vessel was cannulated with a mechanically negligible, size-matched PDMS tube inserted in its lumen as water leakage from Col-HUAs and decellularized Col-HUAs during the test was expected^24^. The vessel was preconditioned by cyclic pressurization between 0 and 150 mmHg ten times. After preconditioning, the vessel was decoupled from the loading frame and recoupled at its updated unloaded configuration (the luminal pressure was about 10 mmHg, and the axial load was about 0 mN). The outer diameter of the vessel was recorded at the unloaded configuration. The axially constrained vessel was then subjected to cyclic pressurization at the unloaded length. Data from the loading phase of the cycle were analyzed for the mechanical properties of the vessel as follows.

The compliance of the vessel was calculated by the Eq. (1).

1 $$ {\text{Compliance (\% per 100 mmHg) = }}\frac{{\left( {D_{1} - D_{2} } \right)}}{{D_{2} \left( {P_{1} - P_{2} } \right)}} \times 10^{4} $$

where $P_{i}$ and $D_{i}$ ($i$ = 1 or 2) respectively represent the transmural pressure and the corresponding outer diameter of the vessel. In this study, the compliance was calculated between 70 and 120 mmHg. That is, $P_{1}$ = 120 mmHg and $P_{2}$ = 70 mmHg. As the compliance can be influenced by the vessel wall thickness, it is more informative to examine the mechanical properties of the HUAs in terms of stress-stretch relationship.

The stress was determined based on the dimensions of the vessel at its deformed state. The unloaded thickness of the vessel, $H$, was measured from the histological section of the vessel using Image J (NIH). The wall volume ($V$) of the vessel was then determined by the Eq. (2),

2 $$ V = \pi \left( {B^{2} - A^{2} } \right)L $$

where $B$ is the unloaded outer radius, $A = (B - H)$ the unloaded inner radius, and $L$ the unloaded length of the HUA.

Although not measurable, the deformed inner radius, $a$, at any deformed state can be computed by the Eq. (3) given the on-line measurement of deformed outer radius, $b$, with the assumption of incompressibility.

3 $$ a = \sqrt {b^{2} - (\frac{{B^{2} - A^{2} }}{{\lambda_{z} }})} $$

where $\lambda_{z} = \frac{l}{L}$ is the axial stretch ratio in which $l$ is the deformed length of the vessel.

Given $a$ and $b$, the mean circumferential stress, $\sigma_{\theta }$, was calculated using the Eq. (4),

4 $$ \sigma_{\theta } = \frac{Pa}{h} $$

where $P$ is the transmural pressure, and $h = (b - a)$ the deformed wall thickness.

The associated mean circumferential stretch ratio, $\lambda_{\theta }$, was determined by the Eq. (5),

5 $$ \lambda_{\theta } = \frac{{{{(a + b)} \mathord{\left/ {\vphantom {{(a + b)} 2}} \right. \kern-\nulldelimiterspace} 2}}}{{{{(A + B)} \mathord{\left/ {\vphantom {{(A + B)} 2}} \right. \kern-\nulldelimiterspace} 2}}} $$

The burst pressure and suture retention strength of the three groups of HUAs were also examined using the same mechanical tester with a 100 psi pressure transducer (Model 80A, 0–100 psi, Sensormate, Taiwan) and a 50 N load cell (MOB-10, Transducer Technique, Temecula, CA) as larger maximum pressure and maximum force were expected. The burst pressure of the HUA, the maximum pressure that the vessel could bear before failure, was measured by gradually increasing the transmural pressure of a preconditioned vessel. Suture retention tests were performed on ~ 20-mm HUA segments with a single 6-0 polypropylene suture placed at 2 mm away from one end of the vessel. The suture was connected to the load cell and the other end of the vessel was fixed to the loading frame. As the vessel was stretched at an extension rate of 0.25 mm/s, the force was recorded until the suture pulled through the vessel.

### Statistical analysis

Data were presented as mean ± standard deviation (SD). The mechanical properties of Unt-HUAs, Col-HUAs, and decellularized Col-HUAs were compared by one-way ANOVA with repeated measures in conjunction with Tukey post-hoc procedure. The collagen and GAG contents of the three HUAs groups as well as the residual DNA in either the Unt-HUAs or the Col-HUAs decellularized under the three decellularization conditions were compared by one-way ANOVA with Tukey post-hoc. The significance level was set at p = 0.05.

## Results

**Figure 2:**
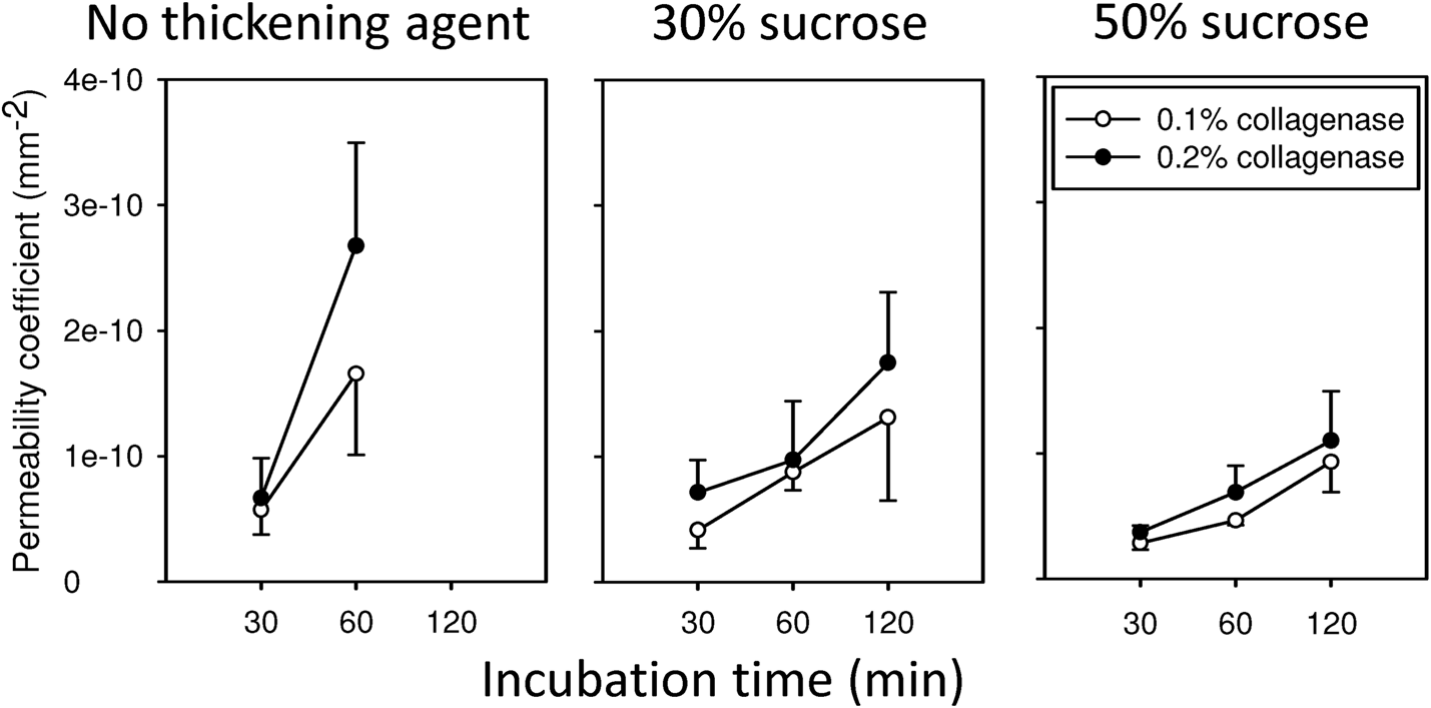
Time courses of permeability changes in the vessel wall of the HUAs treated with the collagenase solution containing no thickening agent (low viscosity), 30% sucrose (medium viscosity), or 50% sucrose (high viscosity). The HUAs treated with the collagenase solution of low viscosity for 120 min leaked tremendously when pressurized thus no permeability coefficient was calculated. Each error bar should be on both sides of the mean; only one side is shown for simplicity.

Figure 2 shows the temporal changes in the permeability of the HUAs treated with the collagenase solution containing no sucrose, 30% sucrose, or 50% sucrose. The collagenase solution containing no sucrose apparently digested the whole vessel wall. Note that 120 min of incubation therein resulted in severe water leakage from the vessels during permeability tests and thus no permeability coefficient was calculated. HUAs remained intact in appearance after being treated with the collagenase solution containing 30% or 50% sucrose for up to 2 h. The collagenase treatment loosened the connective tissue at the abluminal surface of HUAs. This was well illustrated on the H&E-stained sections of Col-HUAs; the abluminal surface of which was smoother than that of Unt-HUAs (Supplementary Fig. 1). Histology also demonstrated that the treatment of the collagenase solution containing sucrose did not damage the media; the collagenase digestion appeared to be localized, limiting its effect to the abluminal surface. Regardless of sucrose concentrations, the permeability coefficients of HUAs increased with the collagenase concentration and duration of digestion. The permeability variations were smaller, in particular, for the HUAs treated with the collagenase solution containing 50% sucrose. Therefore, for all the subsequent experiments involving the abluminal lining removal, HUAs were treated with the collagenase solution containing 50% sucrose for 60 min as the treatment resulted in acceptable vessel permeability with better consistency. When Unt-HUAs were examined for permeability, no water leaked out of the pressurized Unt-HUAs; the permeability coefficient of Unt-HUAs was zero.

**Figure 3:**
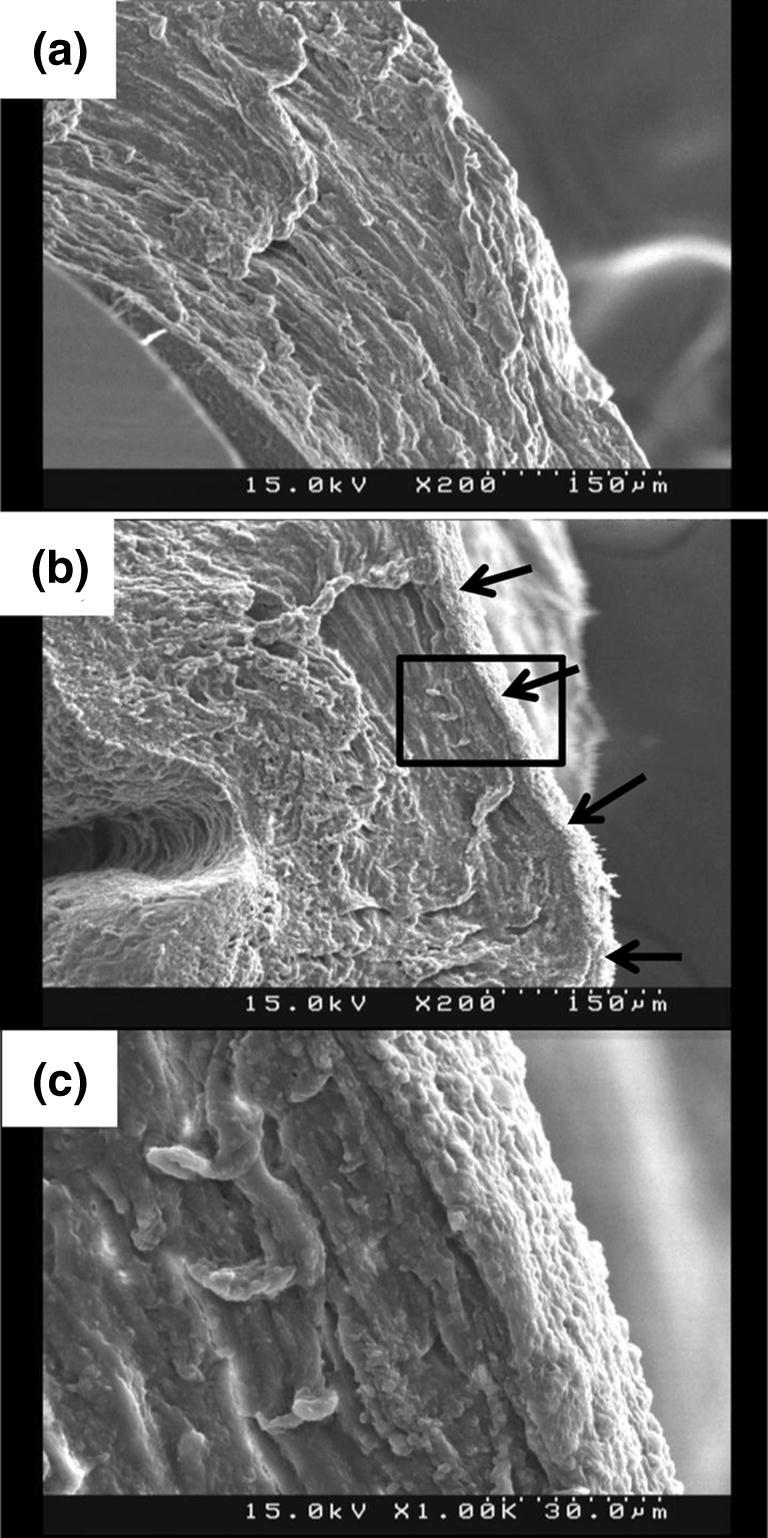
Representative SEM images of Col-HUA (**a**) and Unt-HUA (**b**,**c**) cross-sections. The arrows indicate the microstructure that was absent in Col-HUAs. Panel (**c**) shows higher magnification view of the box outlined in panel (**b**).

**Figure 4:**
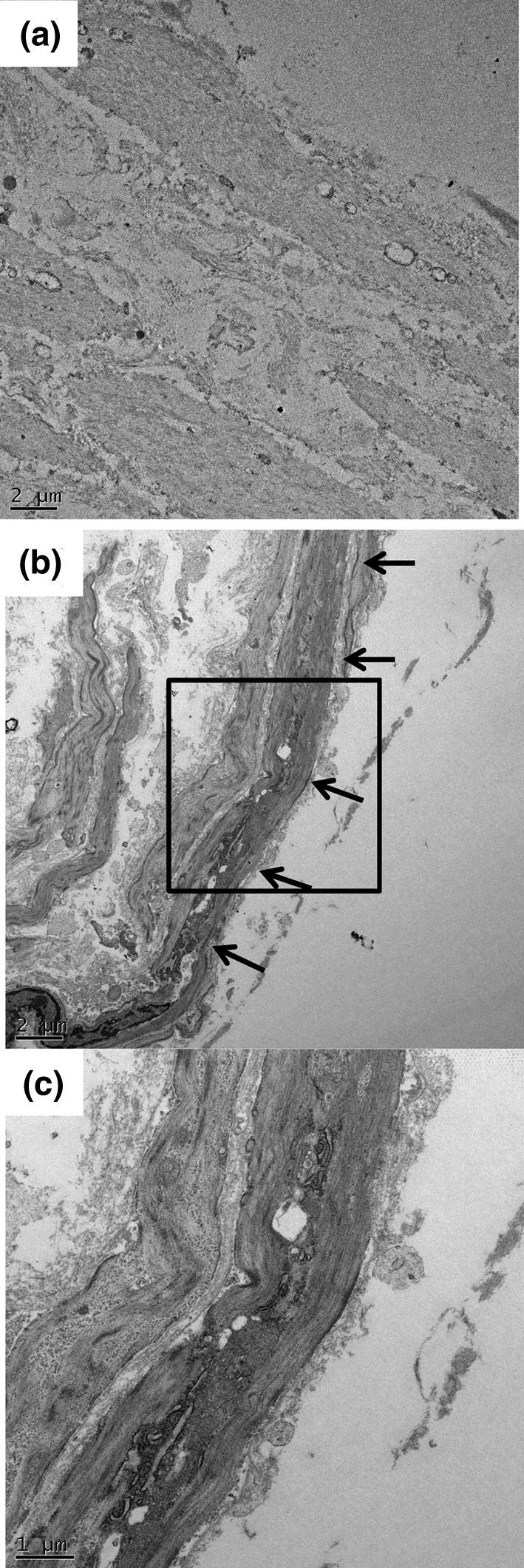
Representative TEM images of Col-HUA (**a**) and Unt-HUA (**b**,**c**) cross-sections near the abluminal surface. The arrows indicate the microstructure that was absent in Col-HUAs. Panel c shows higher magnification view of the box outlined in panel (**b**).

Figures 3 and 4 show, respectively, the representative SEM and TEM images of Col-HUAs and Unt-HUAs near the abluminal surface. The SEM image of the Unt-HUA revealed a protective lining at the abluminal surface, which was absent from the SEM image of the Col-HUA. Note also that the vessel wall of the Col-HUA remained intact, indicating that collagenase digestion was restricted to the abluminal surface. The TEM image of the Unt-HUA cross-section revealed an electron dense lining near the abluminal surface, which was also absent from the mostly electron lucent Col-HUA cross section. In another TEM image (Supplementary Fig. 2), the lining tissue was separated from the underlying tissue probably due to tissue sectioning, suggesting that the lining tissue is structurally distinct from the underlying tissue. Based on TEM observation, the lining appeared to be acellular and had a thickness of 2–3 μm.

**Figure 5:**
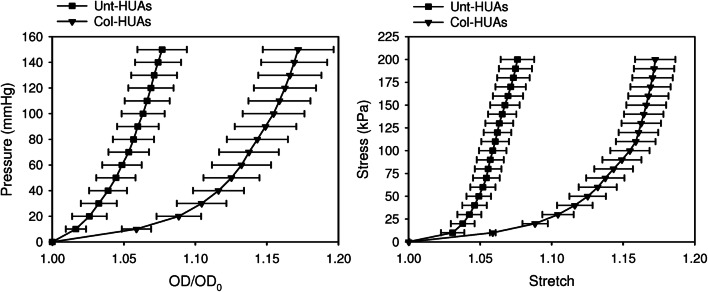
Average pressure-diameter curves (**a**) and circumferential stress-stretch curves (**b**) of Unt-HUAs and Col-HUAs. Data are presented as mean ± SD (n = 5).

Figure 5 shows the mechanical properties of Unt-HUAs and Col-HUAs. Both the pressure-diameter curves (mechanical behavior including both structural and material contributions) and stress-stretch curves (mechanical behavior free of structural contribution) shifted to the right after collagenase treatment. As the collagenase treatment did not change the vessel thickness, the shifting of both curves could be due to either changes in materials or the removal of some structure that has a negligible thickness. Note that the toe region of the stress-stretch curve became more significant and the slope of the stress-stretch curve at the linear region remained unchanged after collagenase treatment. That is, the collagenase treatment did not change the main load bearing capability of HUAs. Together with the histological finding that the media was not affected by collagenase treatment (will be shown below), the changes in mechanical behavior of HUAs suggest the removal of a stiff abluminal lining.

**Figure 6:**
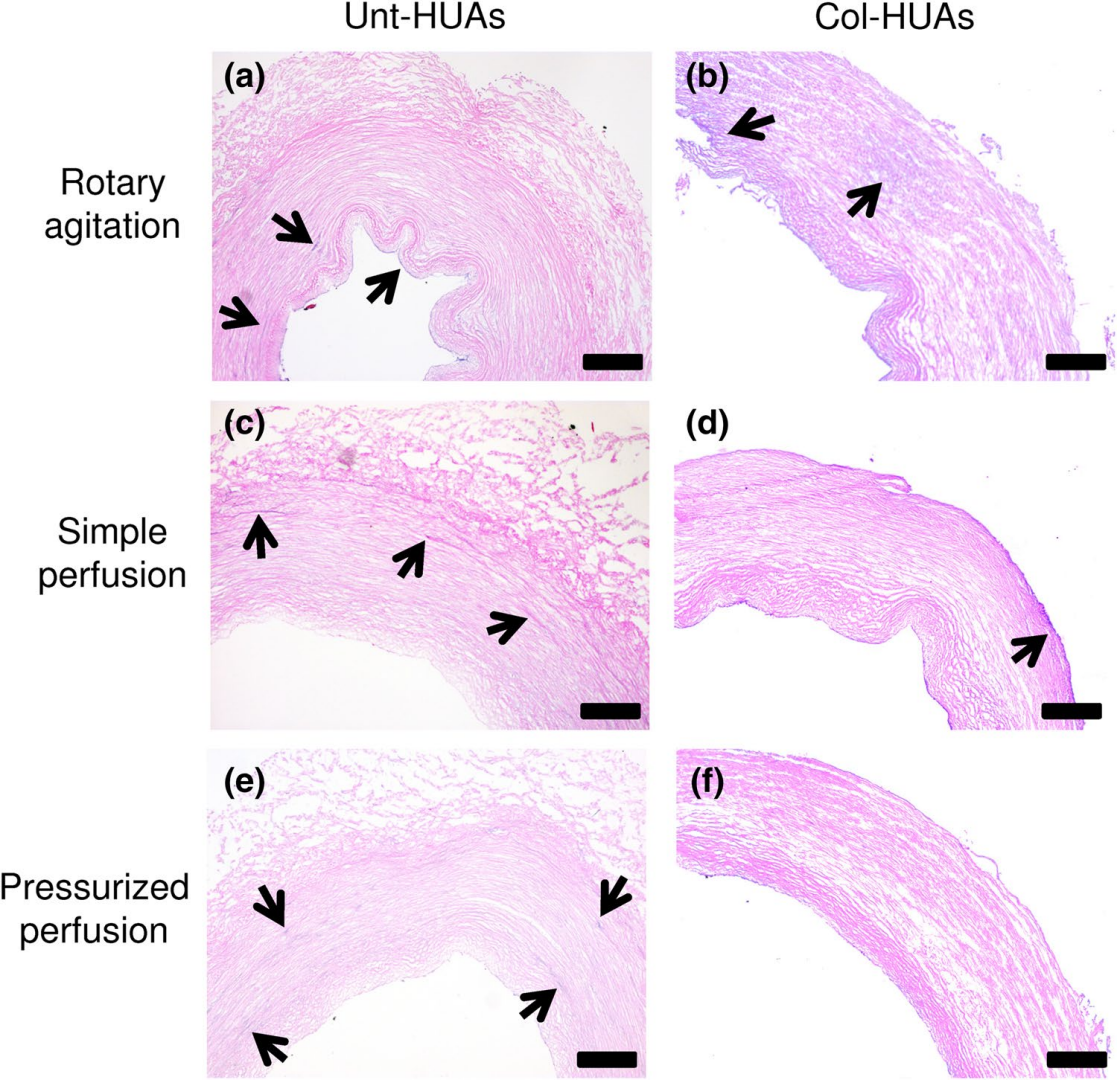
Illustration of residual DNA in Unt-HUAs (**a**,**c**,**e**) and Col-HUAs (**b**,**d**,**f**) decellularized with 1% sucrose solution under rotary agitation, simple perfusion, or pressurized perfusion for 24 h with H&E stained histological sections. Arrows indicate nuclei debris.

Figure 6 shows the representative H&E-stained cross-sections of the Col-HUAs and Unt-HUAs that were decellularized with the SDS solution under rotary agitation, simple perfusion, or pressurized perfusion for 24 h. A few nuclei remained visible on the Unt-HUAs decellularized under simple agitation. While no nucleus was observed on the sections of the Unt-HUAs decellularized under simple perfusion or pressurized perfusion, there appeared blue diffuse smears in their media. The finding is consistent with previous reports^19,25^, in which HUAs were decellularized by surfactants only. The smears were also seen on the sections of the Col-HUAs decellularized under simple agitation whereas they are absent from the sections of the Col-HUAs decellularized under simple perfusion and pressurized perfusion. Simple perfusion, in general, appeared to be as effective as pressurized perfusion based on the histological findings.

**Figure 7:**
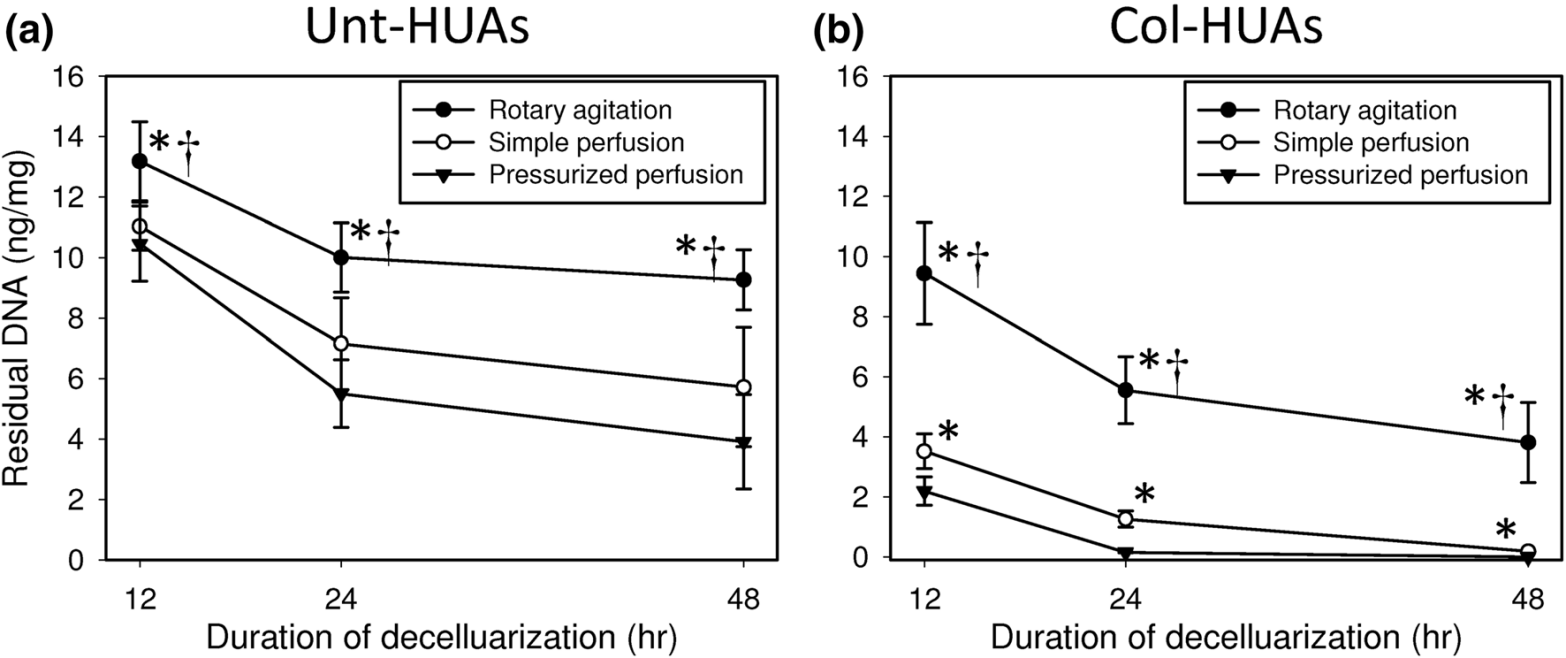
Time course of residual DNA in Unt-HUAs (**a**) and Col-HUAs (**b**) decellularized with 1% SDS solution under rotary agitation, simple perfusion, or pressurized perfusion. Data are presented as mean ± SD (n = 5). *p < 0.025 vs. pressurized perfusion; ^†^p < 0.025 vs. simple perfusion.

The efficiency of decellularization can be more objectively evaluated by quantifying residual DNA in the vessel. Figure 7 shows that the amount of DNA remaining in both Unt-HUAs and Col-HUAs depended on the decellularization conditions in the order: rotary agitation > simple perfusion > pressurized perfusion for all the time points. Regardless of decellularization conditions, however, an appreciable amount of residual DNA remained in the Unt-HUAs decellularized for up to 48 h. When compared with Fig. 7a, Fig. 7b shows that decellularization efficiency was significantly improved upon the abluminal lining removal. Particularly, complete removal of DNA was accomplished within 24 h by pressurized perfusion of the SDS solution. Results of residual DNA quantification were consistent with the histological findings.

**Figure 8:**
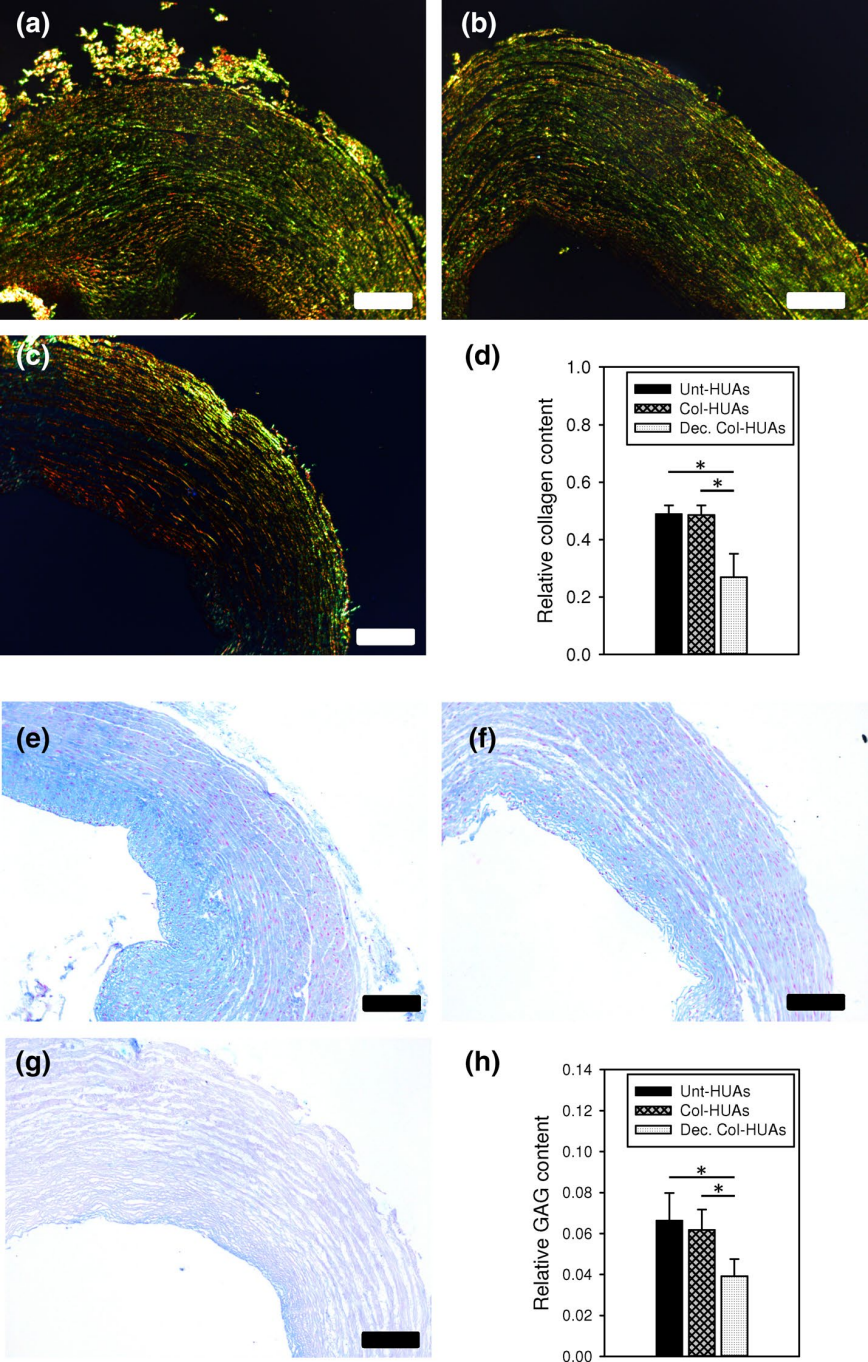
Representative PSR stained (**a**–**c**) and Alcian blue stained (**e**–**g**) of Unt-HUAs (**a**,**e**), Col-HUAs (**b**,**f**), and decellularized Col-HUAs (**c**,**g**). Comparisons of relative collagen (**d**) and GAG (**h**) contents among the three groups. Data are presented as mean ± SD (n = 3). *p < 0.025. Scale bar 100 μm.

Figure 8 shows the representative images of PSR-stained and Alcian blue-stained cross sections of Unt-HUAs, Col-HUAs, and decellularized Col-HUAs and their corresponding semi-quantitative analysis. The PSR images revealed that birefringence intensity on the Col-HUA and Unt-HUA sections was comparable whereas the intensity decreased after decellularization. The corresponding image quantification (Fig. 8d) also showed that the staining intensity on the decellularized CT-HUA sections was significantly lower than that on the Unt-HUA and Col-HUA sections, indicating that the collagen in the media was not affected by collagenase treatment and some collagen was removed by decellularization. Similarly, the Alcian blue staining on the Col-HUA section was comparable to that on the Unt-HUA section and the staining decreased after decellularization, which was verified by image quantification (Fig. 8h); that is, the collagenase treatment did not change the GAGs content but decellularization reduced the GAG content significantly.

**Figure 9:**
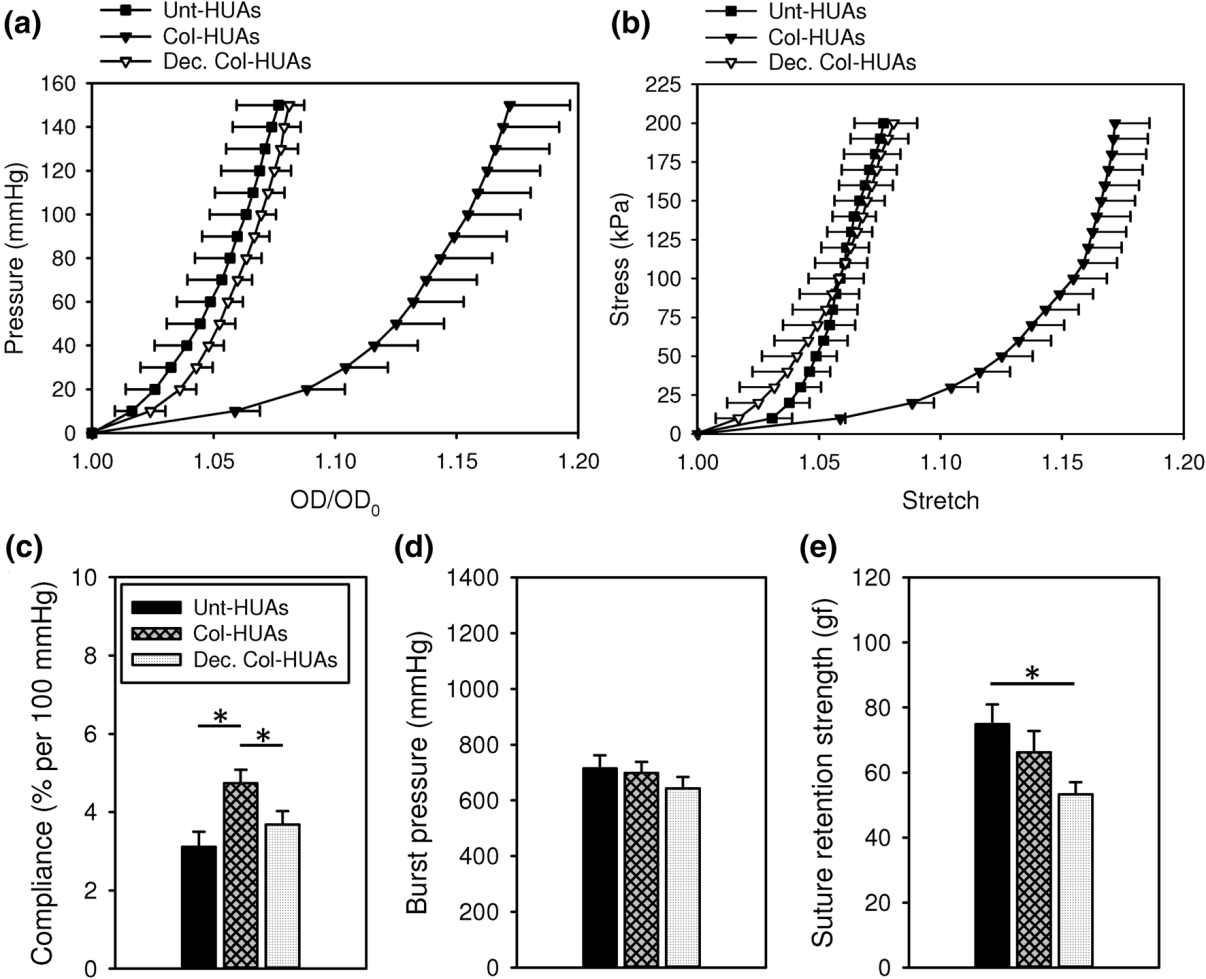
Average pressure-diameter curves (**a**) and circumferential stress-stretch curves (**b**) of Unt-HUA, Col-HUAs and decellularized Col-HUAs. (**c**) Comparisons of the compliance, burst pressure, and suture retention strength among Unt-HUAs, Col-HUAs, and decellularized Col-HUAs. Data are presented as mean ± SD (n = 5). *p < 0.05.

Figure 9 shows both the pressure-diameter and stress–strain curves shifted to the left after decellularization; that is, the ECM components removed by decellularization might contribute to the mechanical behavior of HUAs. Figure 9c show the compliance, burst pressure and suture retention strength of Unt-HUAs, Col-HUAs, and decellularized Col-HUAs. The compliance (i.e., the reciprocal of structural stiffness) of Col-HUAs was significantly greater than that of Unt-HUAs and decellularized Col-HUAs. Note that decellularized Col-HUAs had a compliance similar to that of Unt-HUAs. No significant differences in the burst pressure were found among the three groups of HUAs. The suture retention strength, however, significantly decreased in Col-HUAs and decellularized Col-HUAs compared to that of Unt-HUAs.

## Discussion

We demonstrated for the first time the presence of a watertight barrier at the abluminal side of HUAs by the abluminal collagenase treatment, which rendered HUAs water permeable without damaging the underlying media. The presence of the abluminal lining was further supported by comparing the SEM and TEM images, and the mechanical properties of Unt-HUAs and Col-HUAs. In our pilot study to test if there is a watertight barrier at the luminal side of HUAs, highly viscous collagenase solution was delivered to and confined within the luminal space, allowing the collagenase to work on luminal surface only. Also, HUAs were turned inside-out and the *external* luminal surface was then treated with collagenase. In both cases, the vessel wall of the treated HUAs remained impermeable to water, indicating that the watertight barrier is not at the luminal side of HUAs.

The addition of sucrose, serving as a thickening agent, in collagenase solution increased its viscosity (i.e., reduced its fluidity), which resulted in the spatially restricted activity of collagenase and hence made localized digestion possible. The highly viscous collagenase solution was effective in removing the abluminal lining while preserving the underlying media. The dependence of the vessel permeability on the collagenase concentration and digestion duration indicates that the watertight lining is composed of some substrates of collagenase. In our pilot study, highly viscous hyaluronidase and elastase solutions were also prepared and tested, separately. Neither was effective in making HUAs water permeable, though.

Similar to the adventitia found in other arteries, the abluminal lining appeared to have a specific role in the mechanical behavior of HUAs. The substantial right-shifting of the stress-stretch curve upon collagenase treatment indicates that the abluminal lining may prevent HUAs from over-dilation at low pressure. On the other hand, the independence of the burst pressure on the abluminal lining, indicates that the failure of the abluminal lining may occur earlier than the whole HUA in response to increasing pressure. Although the suture retention strength of HUAs reduced after the abluminal lining removal, it remained sufficient for a successful anastomosis.

The removal of cellular components is essential to prevent severe immune responses for in vivo use of decellularized tissue scaffolds^10,26^. We showed in our previous study that 48 h of decellularization under rotary agitation reduced residual DNA in *short* segments of HUAs to the detection limit of the picoGreen assay^22^. The rotary agitation was not effective for decellularizing *long* segments of HUAs, however. The decellularization of long segments of HUAs may be improved by perfusing decellularization solution into their lumen. Transmural flow that may be introduced by the perfusion, if any, is beneficial to removing immunogenic cellular components in the vessel wall. It was not until the removal of the abluminal lining and hence the associated increase in vessel permeability that the perfusion-assisted decellularization of long segments of HUAs was significantly improved. Residual DNA was almost undetectable by the picoGreen assay in the Col-HUAs decellularized under pressurized perfusion for 24 h. This is a considerable improvement in terms of efficiency for the decellularization of HUAs^19,22,25^ or other blood vessels^27–31^. The improved decellularization efficiency could significantly reduce the cost of production. Additionally, as SDS has been reported to have adverse effects on the ECM, the reduced duration of SDS treatment is favorable. One of the advantages of using decellularized tissue scaffolds for tissue engineering is the availability of bioactive molecules in the scaffold. The reduction in process time may also be beneficial to the preservation of bioactive molecules such as basement membrane components and ECM-bound growth factors (e.g., bFGF^32^ and VEGF^33^). The former could facilitate the recellularization of the vessel with anti-thrombotic endothelial cells as well as smooth muscle cells. The latter could also support reendothelialization and help maintain a functional endothelium.

The convective transmural flow was thought to improve the decellularization of HUVs^21,34–36^. When infused by water, Unt-HUAs were found to be watertight; i.e., no transmural flow. The controversial finding may be due to the difference in nature between HUVs and HUAs. Note, however, that HUVs in their study were trimmed to have a uniform wall thickness by using a cryo lathe prior to decellularization^21^. The machining process could have removed the abluminal watertight lining of HUVs, if any. Nevertheless, an abluminal watertight lining in HUVs was not identified in their studies.

Transmural pressure is expected to decrease gradually along the length of the vessel because of the permeable vessel wall. In our perfusion system, pressure was measured and manipulated downstream from the vessel (see Fig. 1). The pressure, which was set at 30 mmHg for the group of pressurized perfusion, thus ensured transmural flow throughout the entire vessel despite the decreasing transmural flow along the vessel. Histology at three locations of the vessel (upstream, middle, and downstream) showed that no residual nuclei were found at all locations (data not shown). The ECM at the upstream location of vessels that were longer than 6 cm, however, appeared to be condensed after decellularization (data not shown), which might be attributed to relatively higher transmural pressure therein. The reduced vessel permeability and pore size in the condensed ECM might complicate decellularization and future cell seeding, respectively. Because of the pressure drop between upstream and downstream, the length for the whole vessel to be completely decellularized using the current system at the specified conditions without over compression of the ECM is limited (~ 6 cm). Other factors that may cause uneven transmural interstitial flow in the vessel include irregular microstructure and locally varying wall thickness of the vessel. These factors should be borne in mind when decellularizing long tubular tissues by pressurized perfusion.

In vitro recellularization of decellularized blood vessels remains challenging because of their dense microstructure, which hinders cell migration. Shimizu et al. used magnetite nanoparticles to facilitate cell-seeding into decellularized porcine carotid arteries; however, cells were only found on the external surface of the vessel (i.e., no infiltration of cells into the media)^37^. Yazdani et al. showed that the removal of adventitia from decellularized porcine carotid arteries could improve cell seeding efficiency^31^. In their study, the adventitia was mechanically removed, however, which may damage the arterial structure. We attempted to recellularize in vitro the decellularized HUAs in our pilot study. Unfortunately, cells were only found to attach on the external surface of decellularized Col-HUAs without infiltrating into the media (data not shown).

There are a few limitations in this study. We used 1% SDS solution as the decellularizing agent to see if the enhanced permeability benefits the decellularization of HUAs. Other agents (e.g., Triton X-100, and sodium deoxycholate) and their combinations may work better and should be tested for optimal HUA decellularization. It is unclear if the abluminal lining of HUAs has biological roles or physiological functions. The host response of the decellularized Col-HUAs and Unt-HUAs following implantation warrants a detailed study. Note, however, that the different protocols used for decellularizing Col-HUAs and Unt-HUAs, in addition to the presence or absence of the abluminal lining, could lead to different in vivo remodeling outcomes of the two tissues. Because of the many interesting characteristics of the abluminal lining, a detailed proteomic analysis should be conducted to fully understand its composition. Finally, the effectiveness of decellularized Col-HUAs or recellularized Col-HUAs to be used as a vascular graft should be evaluated using vascular reconstructive animal models.

## Conclusion

In this study, we demonstrate the presence of an abluminal watertight lining in HUAs that renders the vessel water impermeable. The removal of the lining without affecting the underlying media was successfully achieved by digesting the abluminal surface of the vessel using a highly viscous collagenase solution. The establishment of transmural flow was verified upon the abluminal lining removal. Evidence from SEM, TEM, and mechanical testing also supported the presence of the abluminal lining. More importantly, the removal of the abluminal lining significantly improved the perfusion-assisted decellularization of HUAs.

## Supplementary information

Supplementary information
