# Supplementary material for: Removal of an abluminal lining improves decellularization of human umbilical arteries
Source: Sci Rep. 2020 Jun 29;10:10556. doi: 10.1038/s41598-020-67417-4 (PMC7324607; doi:10.1038/s41598-020-67417-4)
Supplement: Supplementary file 1 — Supplementary information [file 41598_2020_67417_MOESM1_ESM.pptx]

## Slide 1
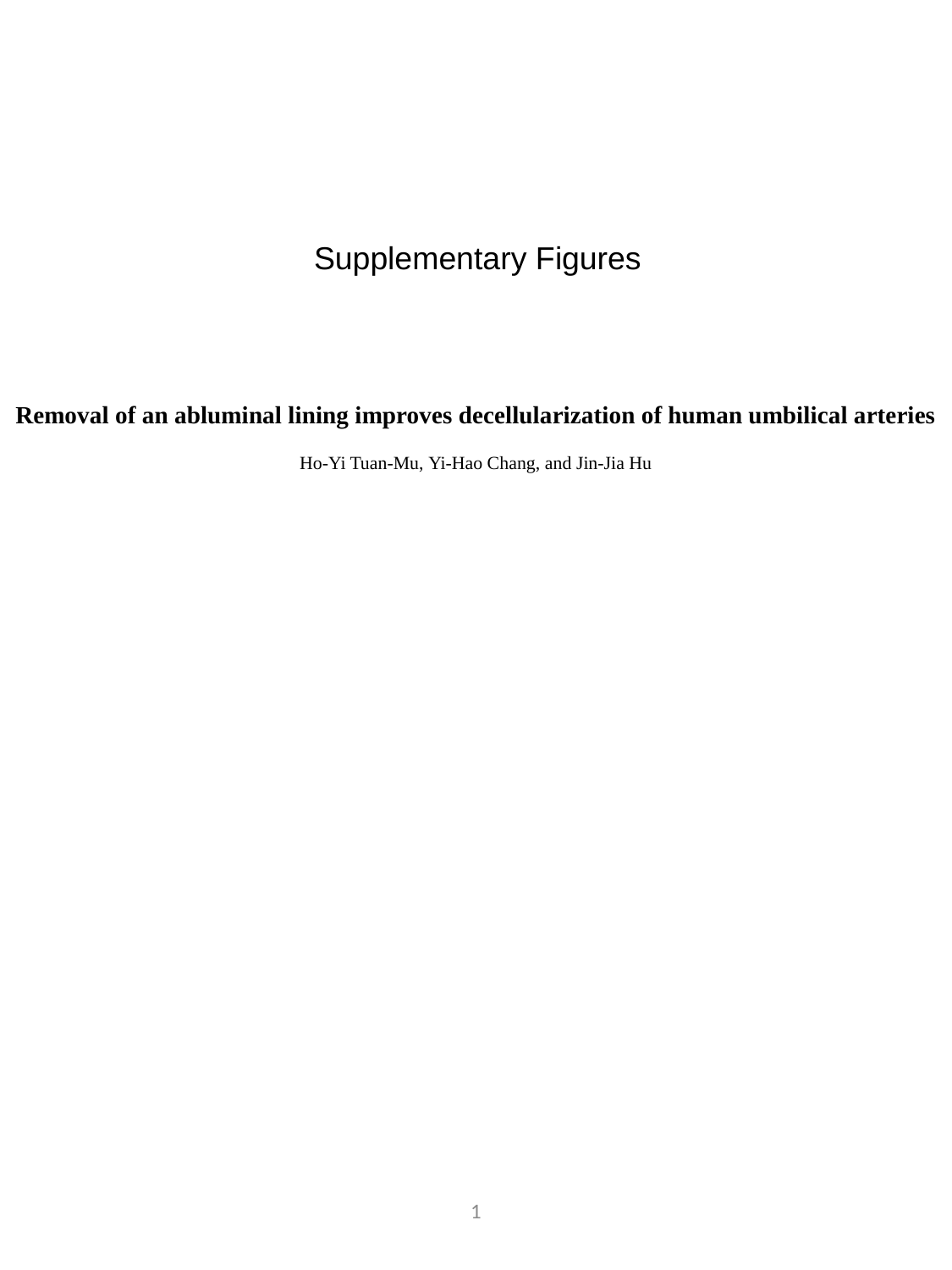

Removal of an abluminal lining improves decellularization of human umbilical arteries
Ho-Yi Tuan-Mu, Yi-Hao Chang, and Jin-Jia Hu
Supplementary Figures
1

## Slide 2
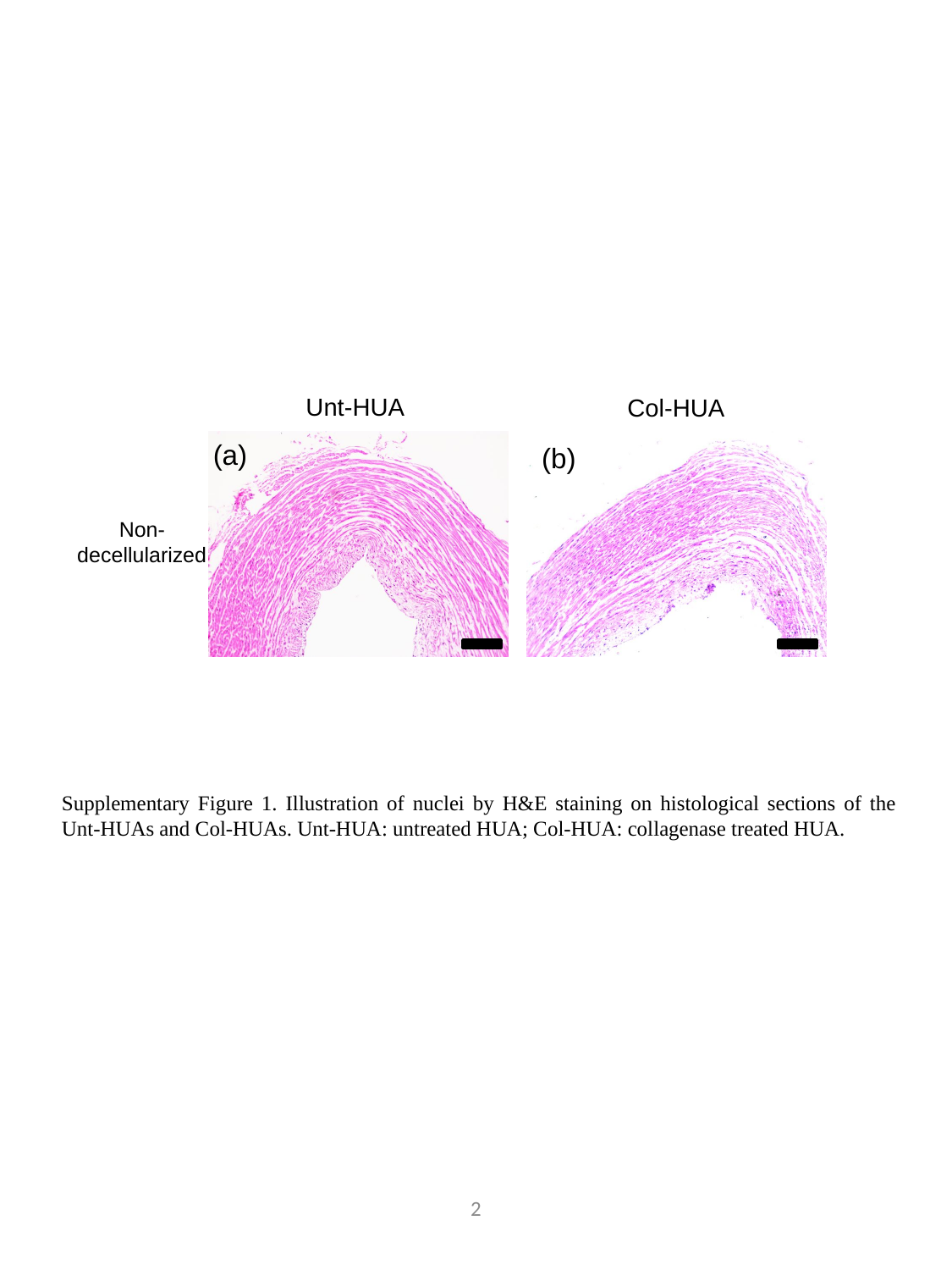

Unt-HUA
Col-HUA
(a)
(b)
Non-decellularized
Supplementary Figure 1. Illustration of nuclei by H&E staining on histological sections of the Unt-HUAs and Col-HUAs. Unt-HUA: untreated HUA; Col-HUA: collagenase treated HUA.
2

## Slide 3
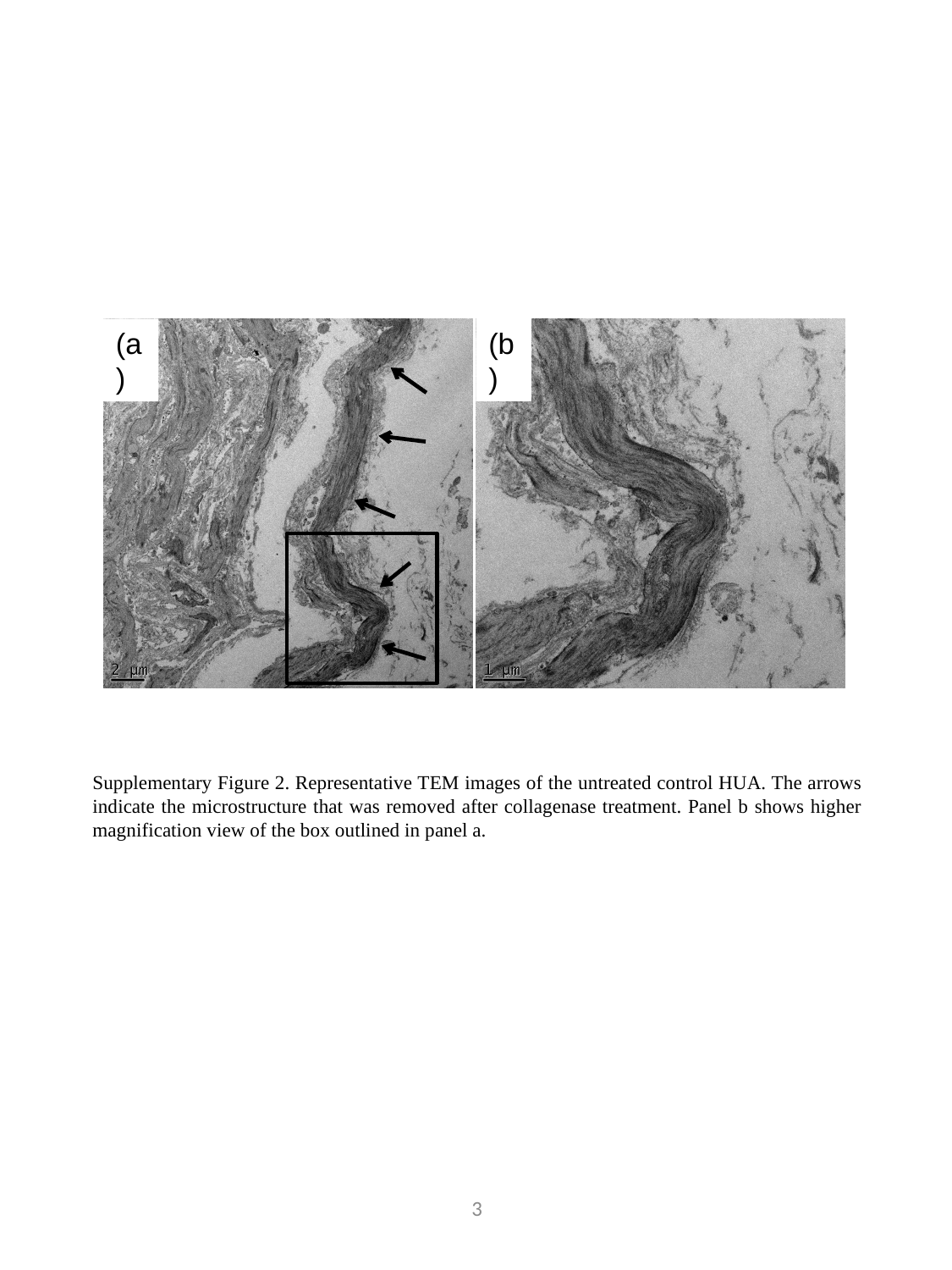

(b)
(a)
Supplementary Figure 2. Representative TEM images of the untreated control HUA. The arrows indicate the microstructure that was removed after collagenase treatment. Panel b shows higher magnification view of the box outlined in panel a.
3
